# Integrated ATAC‐seq and RNA‐seq analysis identifies key regulatory elements in NK cells activated with feeder cells and IL‐2

**DOI:** 10.1002/btm2.10747

**Published:** 2025-02-26

**Authors:** Pedram Motallebnejad, Zion Lee, Jennifer L. One, Frank Cichocki, Wei‐Shou Hu, Samira M. Azarin

**Affiliations:** ^1^ Department of Chemical Engineering and Materials Science University of Minnesota Minneapolis Minnesota USA; ^2^ Department of Medicine University of Minnesota Minneapolis Minnesota USA

**Keywords:** ATAC‐seq, feeder cells, NK cells, super‐enhancers, transcription factor

## Abstract

Natural killer (NK) cells are in development for allogeneic immunotherapy; however, for such use as off‐the‐shelf medicines, NK cells need to undergo ex vivo expansion, typically through activation with feeder cells and cytokines, to generate sufficient cells for clinical applications. Upon stimulation with feeder cells in the presence of cytokines, NK cells undergo profound changes in gene expression, altering their metabolic activity, cell cycle progression, and growth behavior, but the precise changes that drive this transformation remain poorly understood. In this study, we identified significant differences in the transcriptome and chromatin accessibility of NK cells 7 days after feeder cell and cytokine activation, with the changes even more pronounced in genome regions closer to enhancers. Several transcription factors, including AP‐1, IRF4, STATs, T‐bet, Eomes, and bHLHE40, which play key roles in NK cell development and immune response, exhibited differential binding activity between unstimulated and day 7 NK cells. Gene sets composed of target genes downstream of these transcription factors were also enriched at day 7, implying their involvement in NK cell activation. Moreover, we compared potential super‐enhancer regions in NK cells before and after activation, combined with the transcriptional activity of nearby genes. We identified stable and transcriptionally active super‐enhancers in unstimulated and day 7 NK cells, as well as those that form or disappear after co‐culture initiation. The transcriptomic and epigenetic characterization of NK cells presented in this study could facilitate the ex vivo expansion and engineering of functionally superior NK cells.

**Translational Impact Statement**

Expansion of natural killer (NK) cells with feeder cells and cytokines has been widely practiced in clinical applications. Here, we demonstrated the key transcription factors with increased activity in feeder cell‐stimulated NK cells, offering potential strategies for improving the expansion of NK cells. Additionally, we identified stable, transcriptionally active super‐enhancers in feeder cell‐activated and unstimulated NK cells. These sites can be used for targeted insertion of transgenes to achieve stable expression. Our findings hold promise for advancing NK cell therapies with improved functionalities in clinical settings.

## 1 INTRODUCTION

Natural killer (NK) cells are emerging as a major cell type used in cell‐based immunotherapies and have shown promising outcomes in clinical trials for certain cancers.^1^ As part of the innate immune system, NK cells surveil the body for transformed, infected, or stressed cells and eliminate them.^2^ The effector function of NK cells relies on an array of activating and inhibitory receptors that bind to the ligands on target cells to differentiate abnormal cells from healthy cells.^2^ Upon recognizing target cells, NK cells establish immunological synapses on the contact points with the target cell and execute their cytotoxic response by releasing cytolytic granules and inflammatory cytokines.^3^

For immunotherapies, NK cells pose a low risk of inducing cytokine release syndrome, neurotoxicity, and graft‐versus‐host disease.^4^ NK cells can be sourced from various origins, such as peripheral blood, umbilical cord blood, and induced pluripotent stem cells.^5^ Isolation from peripheral or umbilical cord blood typically yields limited numbers of NK cells. Ex vivo expansion of NK cells allows for larger doses to be administered to more patients.^6^ The expanded NK cells can potentially be cryopreserved for use in allogeneic cell therapy. An effective method for NK cell expansion involves co‐culture with engineered K562 feeder cells expressing membrane‐bound IL‐21 (mbIL‐21) and mb4‐1BBL.^7^, ^8^, ^9^ Such NK cells expanded through co‐culture with feeder cells have been used in clinical trials.^8^ Following activation with feeder cells, NK cells exhibit increased growth rates and metabolic activity and undergo genome‐wide transcriptional changes.^10^, ^11^, ^12^ However, the impact of feeder cell activation on chromatin accessibility of NK cells remains largely unexplored.

Cell state transitions in immune cells involve changing patterns of transcription factor (TF) binding to genome regions that regulate the expression of downstream genes. It also entails epigenetic changes mediated by histone modifications, which alter chromatin accessibility to transcription.^13^, ^14^ The alterations of chromatin accessibility help guide the transition of T cells and NK cells from naïve to effector and memory cells.^15^, ^16^ Understanding the changes in the chromatin state of NK cells resulting from stimulation with feeder cells and cytokines provides important insights into the mechanisms of feeder cell‐mediated NK cell activation and may facilitate the manufacturing of NK cell products.

Assay for Transposase‐Accessible Chromatin with Sequencing (ATAC‐seq) is widely used for characterizing chromatin accessibility across the genome. It provides a comprehensive assessment of accessibility across the entire regulatory element landscape, offering important information on the role of different TFs in cell state transitions.^17^ ATAC‐seq has also been used to identify potential super‐enhancer (SE) regions—clusters of enhancers located in proximity that exert a more substantial influence on transcription than regular enhancers.^18^, ^19^, ^20^

In this study, we characterized gene expression and chromatin accessibility changes in NK cells before (unstimulated) and after (day 7) activation through co‐culture with feeder cells in the presence of IL‐2. We found significant alterations in transcriptome and chromatin accessibility. Additionally, we identified several key TFs that displayed notable changes in motif accessibility, footprint depth, and enrichment of gene sets representing their target genes in day 7 NK cells. Potential SEs were identified in each condition and categorized as common to both days or exclusive to either unstimulated or day 7. Integrating these SE regions with RNA‐seq data revealed stimuli‐dependent SEs near activation‐related genes and stable SEs near genes crucial for NK cell identity and function.

## 2 MATERIALS AND METHODS

### 2.1 Cell culture and NK cell co‐culture with feeder cells

K562 cells expressing mb‐4‐1BBL and mb‐IL‐21 (referred to as feeder cells, mb stands for membrane‐bound) were cultured in RPMI 1640 medium containing 10% fetal bovine serum. Feeder cells were irradiated at 10,000 rads, cryopreserved, and thawed for co‐culture with NK cells. Peripheral blood mononuclear cells (PBMCs) were extracted from leukocyte reduction system chambers using Ficoll‐Paque Premium (Cytiva). NK cell enrichment was carried out with the EasySep Human NK Cell Enrichment Kit. NK cells were cultured overnight in B0 medium supplemented with 100 U/mL of IL‐2 (R&D Systems) for recovery. B0 medium is a 2:1 (vol:vol) mixture of DMEM with 4.5 g/L glucose, L‐glutamine, and sodium pyruvate (Gibco) and Ham's F12 Medium (Corning) supplemented with 20 μM 2‐mercaptoethanol (Gibco), 50 μM ethanolamine (Sigma), 10 μg/mL ascorbic acid (Sigma), 1.6 ng/mL sodium selenite (Sigma), 100 U/mL penicillin/streptomycin (Gibco), and 20% heat‐inactivated human AB serum (Valley Biomedical).

### 2.2 Flow cytometry of expanded NK cells

NK cells were stained with Fixable Viability Dye eFluor780 (Thermo Fisher Scientific) for 10 min at 4°C. Next, cells were washed with flow cytometry buffer (FACS) buffer (PBS supplemented with 2% FBS and 2 mM EDTA) prior to staining with antibodies against CD56 (Biolegend; Clone: HDCD56) and CD45 (Biolegend; Clone: HI30) for 20 min at 4°C in the dark. Cells were then washed with FACS buffer and fixed in 2% paraformaldehyde (PFA) prior to flow cytometry analysis.

### 2.3 RNA‐seq sample processing and data analysis

Total RNA from samples was purified using the Qiagen™ RNeasy Mini Kit. The Illumina TruSeq® Stranded mRNA Sample Prep Kit protocol was employed to isolate poly‐A containing mRNA molecules and generate sequence libraries. Pooled libraries were sequenced using the Illumina HiSeq 2500 at the University of Minnesota Genomics Center.

Fastq files were mapped to the human genome (hg38) using STAR, and raw counts were obtained with HT‐seq. Fragments Per Kilobase of transcript per Million mapped reads (FPKM) values were computed using Cufflinks and normalized to transcripts per million (TPM) values in R. Genes expressed in at least three samples with counts per million (CPM) > 1 were retained for analysis (14,790 genes). Filtered genes underwent TMM normalization for PCA and clustering in R. EdgeR identified differentially expressed genes (DEGs) at day 7 versus unstimulated, with differential expression criterion of |log_2_(fold change)| > 1 and adjusted *p* value <0.05.

Overrepresentation analysis (ORA) was performed using the ClusterProfiler package^21^ to identify functional groups among downregulated and upregulated DEGs. Gene sets from Reactome, KEGG, Gene Ontology Biological Processes (GO BP), GO Molecular Functions (GO MF), and GO Cellular Components (GO CC) were employed. Gene set enrichment analysis (GSEA) utilized the GSEA software with log_2_TPM data and the list of target genes for each TF as inputs.

### 2.4 ATAC‐seq sample processing and data analysis

Samples comprised of 50,000 cells were processed following the OMNI‐ATAC protocol,^22^ and transposed DNA was purified using the MinElute PCR cleanup kit. Illumina NovaSeq 6000 generated >50 million paired‐end reads after library preparation. Trimmomatic trimmed adapter sequences and low‐quality base calls. Reads were mapped to the human reference genome (hg38) using Bowtie2 (parameters: p 2 ‐X 2000 –very‐sensitive), excluding mitochondrial reads, and deduplicated using MarkDuplicates. MACS2 was used to perform peak calling (parameters: −p 0.01 –nomodel –shift −75 –extsize 150 –bdg), and IDR was used to create a reproducible peak list for unstimulated and day 7 samples. Consensus peaks were determined as the union of unstimulated and day 7 peaks. Raw peak count matrix was obtained using featureCounts. For differential accessibility, GC content bias was corrected using EDASeq, and CPM values were TMM normalized using EdgeR. Normalized ATAC peak counts were used for PCA analysis and clustering in R. EdgeR identified differentially accessible peaks for day 7 versus unstimulated samples, with a criterion of |log_2_(fold change)| > 1 and adj. *p* value < 0.05.

### 2.5 Transcription factor binding motif accessibility and footprint analyses in NK cells

To assess changes in TF binding motif (TFBM) accessibility resulting from NK cell activation with feeder cells and IL‐2, we utilized the chromVAR package in R with TFBM information from HOMER.^23^ ChromVAR calculates a “raw accessibility deviation” for each TFBM, representing the difference between the mapped reads to the TFBMs encompassing peaks and the expected reads based on the average of all samples. It generates a “background” peak set with matching GC content and computes average accessibility and raw background accessibility deviation. These deviations are used to calculate a bias‐corrected deviation and z‐score for each TFBM and sample, indicating the gain or loss of accessibility relative to the average sample profile.

TOBIAS footprint analysis was utilized to visualize the footprints around the TFBMs.^24^ Following the TOBIAS pipeline, the aggregate footprint signals for a TFBM are plotted versus the distance from the TFBM center in order to visualize the aggregated footprint depth and accessibility around the TFBM throughout the genome.

### 2.6 Identifying potential super‐enhancers

To identify SEs, aligned ATAC‐seq reads were combined to create a bam file for each time point. The combined files for each time point were subjected to MACS2 peak calling with the following settings: −p 0.00001 –nomodel –shift −100 –extsize 200 –bdg. Subsequently, using HOMER, peaks situated within 1 kb upstream and 100 bp downstream of a transcription start site (TSS) were excluded. BEDTools package was utilized to merge adjacent peaks separated by less than 12.5 kb. The read coverage of merged peaks was determined, and the background coverage derived from NK cell input DNA sequencing (GEO accession number GSM2048316) within the identical genomic areas was subtracted to obtain the ATAC signal, as described before.^19^ The merged peaks for unstimulated and day 7 NK cells were ranked based on their ATAC signal values, and the inflection point in the ATAC signal versus rank curve was used as the threshold to distinguish regular enhancers from super‐enhancers, as previously described.^25^

## 3 RESULTS

### 3.1 Broad transcript changes upon feeder cell‐mediated activation

**FIGURE 1:**
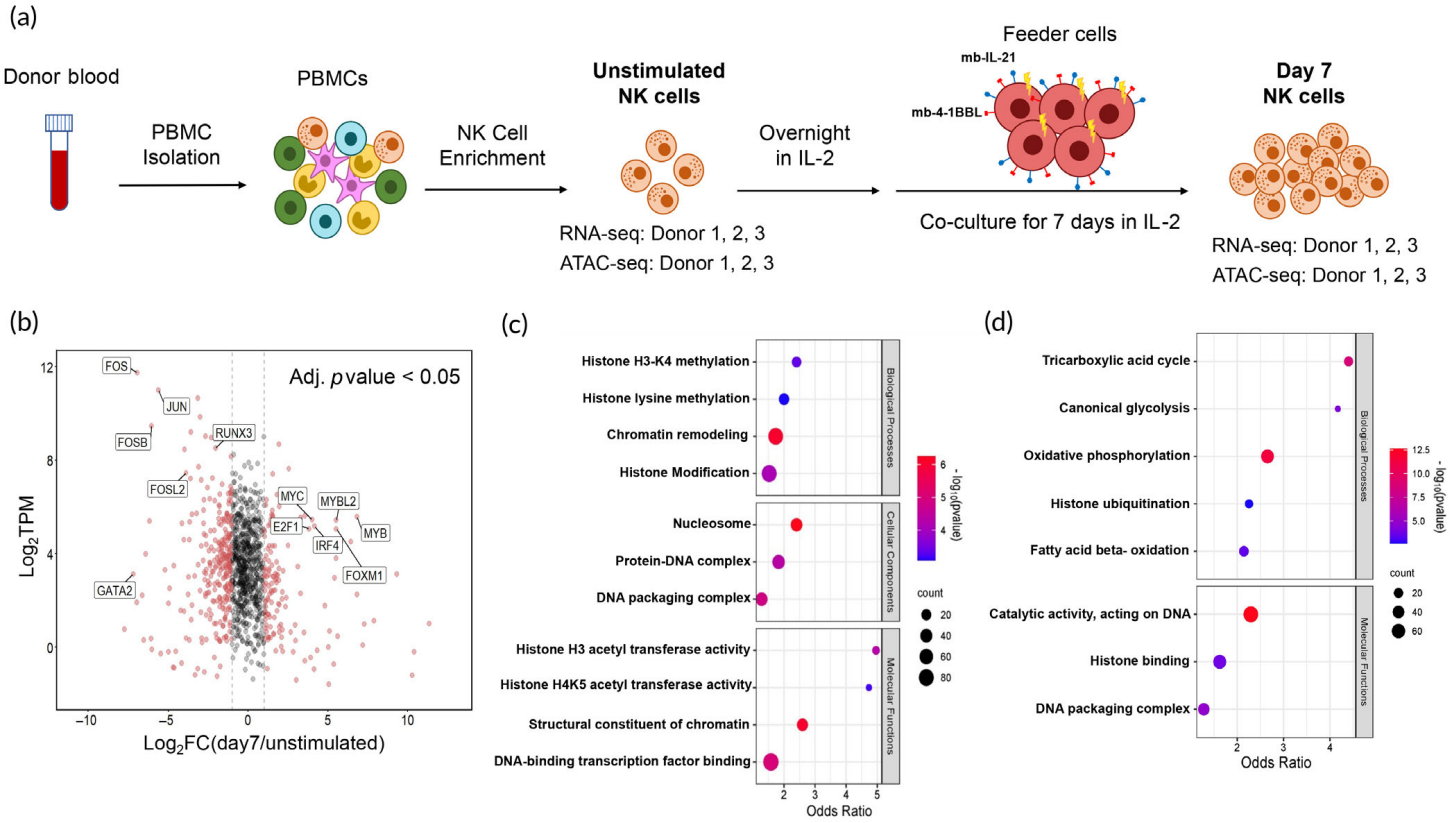
RNA‐seq results suggest changes in chromatin state of NK cells. (a) A schematic illustrating NK cell activation by feeder cells and IL‐2. (b) Average transcript abundance (*n* = 3, in TPM) of all TFs is plotted against the fold change in transcript expression. The dashed lines indicate FC = 2 and FC = 1/2. Some of the TFs with critical roles in immune response or cell proliferation are labeled. Red denotes DEGs. (c, d) Enriched functional classes in downregulated (c) and upregulated (d) DEGs by over‐representation analysis using gene ontology (GO) biological processes, GO cellular components, and GO molecular function data sets. The color bar represents −log_10_(*p* value), and the number of genes in each group is indicated by the size of the circle.

Enriched NK cells from PBMCs were cultured in media containing IL‐2 overnight. Subsequently, NK cells were co‐cultured with irradiated feeder cells at 2 × 10^5^ cells/mL and 4 × 10^5^ cells/mL, respectively, in B0 media with IL‐2 supplementation for 7 days (Figure 1a). Samples were collected right after enrichment (unstimulated) and 7 days after co‐culture initiation (day 7) for RNA‐seq and ATAC‐seq. To assess the purity of day 7 samples, CD45 and CD56 expression on NK cells were evaluated using flow cytometry. The results showed that ≥96% of the cells were positive for these markers (Figure S1a). Additionally, transcript expression levels of various PBMC lineage markers indicated minimal contamination from other PBMCs, such as B cells, T cells, and monocytes, in both unstimulated and day 7 samples (Figure S1b–d). These samples expressed NK cell markers at levels consistent with pure NK cells (Figure S1e). Furthermore, leukemia marker expression was undetectable across all samples, confirming the absence of feeder cells (Figure S1f).

NK cell fold expansion at day 7 showed minimal differences among the three donors (Figure S2a). The transcript expression levels of activating and cytokine receptors, shown in Figure S2b–e, indicate no significant differences between donors, suggesting that the activation degree observed in these donors is similar. Figure S3a,b summarizes the general characteristics of the transcriptome. The hierarchical clustering tree reveals sample clustering at the time of collection (Figure S3a). The PCA plot illustrates that PC1 (accounting for 93% of the variance) effectively distinguishes unstimulated from day 7 samples (Figure S3b). Differential expression analysis of day 7 and unstimulated samples identified a total of 6617 DEGs, with 3299 upregulated and 3318 downregulated (Figure S3c).

### 3.2 Differentially expressed TFs in activated NK cells

TFs play key roles in the activation of NK cells and elicit broad changes in gene expression.^26^ A comprehensive list of human TFs from Lambert et al.^26^ was used to plot their average transcript abundance in all the samples against transcript fold change (day 7/unstimulated) (Figure 1b). In the day 7/unstimulated comparison, 140 TFs were upregulated, while 277 were downregulated. Some of the upregulated TFs were associated with proliferation and metabolic activities, including *MYC*, *IRF4*, *E2F*s, *FOXM1*, and *MYBL2*,^27^, ^28^, ^29^, ^30^, ^31^ but some immune response‐related TFs such as *RUNX3*, *FOS*, *FOSB*, and *FOSL2* were downregulated (Figure 1b).^32^, ^33^, ^34^ Some of the differentially expressed TFs, including *RUNX3*, *MYB*, *GATA2*, *IRF4*, and *JUN* and *FOS* dimers, belong to a group called pioneering TFs, which can bind to condensed chromatin and displace nucleosomes to increase chromatin accessibility.^35^, ^36^ Changes in the expression of pioneering TFs suggest potential alterations in chromatin accessibility of NK cells upon activation by feeder cells and IL‐2.

### 3.3 Functional enrichment of downregulated DEGs: Histone, nucleosome, and DNA binding

ORA on all the upregulated and downregulated DEGs using GO functional classes was performed. Gene description and transcript expression information for some of the notable genes in the enriched gene sets are summarized in Table 1. Enrichment in several histone and chromatin modification gene sets was seen in the transcripts downregulated at day 7 (Figure 1c), including two histone methylation gene sets, histone lysine methylation and histone H3‐K4 methylation. DEGs in these gene sets included methyltransferases and demethylases as shown in Table 1.

**TABLE 1:** List of genes discussed in overrepresentation analysis (ORA) with their description and expression information.

Gene symbols	Functional classes	Gene description	Average TPM	Log_2_FC (d7/us)
*SETD1A*	Histone lysine methylation	SET domain containing 1A, histone lysine methyltransferase	17.41	−1.18
*SETD1B*	Histone lysine methylation	SET domain containing 1B, histone lysine methyltransferase	13.70	−1.85
*ASH1L*	Histone lysine methylation	ASH1‐like histone lysine methyltransferase	12.14	−1.25
*KMT2B*	Histone lysine methylation	Lysine methyltransferase 2B	38.85	−1.03
*KMT2D*	Histone lysine methylation	Lysine methyltransferase 2D	40.18	−2.09
*KDM6A*	Histone lysine methylation	Lysine demethylase 6A	23.07	−1.26
*KDM6B*	Histone lysine methylation	Lysine demethylase 6B	12.19	−2.78
*SETBP1*	Histone lysine methylation	SET binding protein 1	6.54	−2.07
*H1‐3*	Nucleosome and DNA packaging	H1.3 linker histone, cluster member	22.43	−4.72
*H3‐3B*	Nucleosome and DNA packaging	H3.3 histone B	2510.64	−2.78
*H2BC5*	Nucleosome and DNA packaging	H2B clustered histone 5	33.88	−3.39
*H1‐4*	Nucleosome and DNA packaging	H1.4 linker histone, cluster member	62.88	−6.76
*EP300*	Protein‐DNA complex	E1A binding protein p300	30.11	−1.59
*KAT6A*	Protein‐DNA complex	Lysine acetyltransferase 6A	30.69	−1.16
*KAT6B*	Protein‐DNA complex	Lysine acetyltransferase 6B	26.56	−1.10
*KDM5A*	Histone acetyltransferase activities	Lysine methyltransferase 5A	34.71	−1.43
*KAT7*	Histone acetyltransferase activities	Lysine acetyltransferase 7	50.49	−1.30
*KAT2B*	Histone acetyltransferase activities	Lysine acetyltransferase 2B	48.12	−1.80
*ARID5A*	Histone acetyltransferase activities	AT‐rich interaction domain 5A	68.13	−1.81
*HDAC4*	Histone Deacetylase Activity	Histone deacetylase 4	8.38	−1.71
*HDAC5*	Histone Deacetylase Activity	Histone deacetylase 5	19.56	−1.16
*SIRT1*	Histone Deacetylase Activity	Sirtuin 1	12.85	−1.41
*SIRT7*	Histone Deacetylase Activity	Sirtuin 7	51.42	−1.19
*PKM*	Canonical glycolysis	Pyruvate kinase M1/2	959.82	4.14
*ENO1*	Canonical glycolysis	Enolase 1	2056.45	4.03
*PFKM*	Canonical glycolysis	Phosphofructokinase, muscle	10.41	1.84
*HK1*	Canonical glycolysis	Hexokinase 1	49.71	1.62
*HK2*	Canonical glycolysis	Hexokinase 2	12.10	5.52
*CS*	Tricarboxylic acid cycle	Citrate synthase	105.00	1.08
*ACO1*	Tricarboxylic acid cycle	Aconitase 1	5.75	1.60
*IDH3A*	Tricarboxylic acid cycle	Isocitrate dehydrogenase (NAD(+)) 3 catalytic subunit alpha	30.63	1.79
*OGDH*	Tricarboxylic acid cycle	Oxoglutarate dehydrogenase	36.45	1.02
*SDHA*	Tricarboxylic acid cycle	Succinate dehydrogenase complex flavoprotein subunit A	131.10	1.13
*CPT2*	Fatty acid beta‐oxidation	Carnitine palmitoyltransferase 2	9.02	2.08
*ACADM*	Fatty acid beta‐oxidation	Acyl‐CoA dehydrogenase medium chain	35.47	2.33
*HADHA*	Fatty acid beta‐oxidation	Hydroxyacyl‐CoA dehydrogenase trifunctional multienzyme complex subunit alpha	86.30	1.35

Among GO cellular components terms, nucleosome and DNA packaging were enriched. DEGs in these GO terms include histone subunits, which constitute nucleosomes and impact nucleosome stability and chromatin compaction.^37^ Additionally, the protein‐DNA complex GO term was enriched, encompassing several differentially expressed TFs, and histone subunits, along with acetyltransferases and methyltransferases (Table 1).

The enriched GO terms of molecular functions include histone H3 and H4K5 acetyltransferase activities, encompassing several histone acetyltransferase and histone deacetylase genes (Table 1). Acetylation of histones reduces their positive charge, making them less likely to interact with the negatively charged DNA, and results in an open or relaxed chromatin structure.^38^ Overall, ORA revealed profound changes in gene sets related to histone modification and nucleosome and DNA packaging, suggesting alterations in chromatin accessibility upon NK cell activation by feeder cells and IL‐2.

By incorporating additional gene sets from Reactome and KEGG for ORA, multiple functional groups capturing activating receptor signaling and immune response, cytokine and chemokine signaling, as well as cytoskeletal and actin organization were identified as enriched in downregulated DEGs (Figure S3d).

### 3.4 Upregulated functional classes: Metabolism and histone binding

Several functional groups related to metabolism, including glycolysis, the TCA cycle, oxidative phosphorylation, and fatty acid beta‐oxidation, were overrepresented among upregulated DEGs (Figure 1d). Upregulated genes in these pathways include *PKM, ENO1, PFKM, HK1*, and *HK2* in glycolysis; *CS, ACO1, IDH3A, OGDH*, and *SDHA* in the TCA cycle; and *CPT2, ACADM, HADHA*, and *HADHB* in fatty acid beta‐oxidation (Table 1). Upregulation of energy metabolic pathways is crucial for NK cell transition to a highly proliferative state, providing necessary energy and biosynthetic precursors for cell growth. These pathways also supply metabolic intermediates to serve as donors for methyl, acetyl, and other histone acylation groups to facilitate histone modification.^39^ Acetyl‐CoA is an initiating metabolite of the TCA cycle, and its concentration is affected by TCA cycle activity.^40^ Glycolysis flux also quantitatively mediates specific histone acetylation sites and influences the activation of T cells.^41^, ^42^, ^43^, ^44^ α‐Ketoglutarate, a TCA cycle intermediate, participates in the DNA demethylation reaction.^45^ Fatty acid oxidation and metabolism is also a major source of intermediate metabolites like acetyl‐CoA, butyryl‐CoA, and crotonyl‐CoA, which could modify histones.^46^, ^47^ Other GO functional classes enriched at day 7 include histone ubiquitination, catalytic activity acting on DNA, histone binding, and DNA packaging complex, all of which are related to chromatin remodeling. There are not any gene sets related to TF‐DNA interactions and DNA packaging belonging to cellular components, and thus this functional class is not included in Figure 1d.

Additional enriched classes among upregulated DEGs include those associated with cell cycle, DNA replication, and telomere lengthening (Figure S3e). Given the accelerated growth in activated NK cells, upregulation of these pathways was anticipated. The enrichment of pathways related to telomere lengthening suggests a potential mechanism to prevent senescence in NK cells.

### 3.5 Extensive alterations in chromatin accessibility of activated NK cells

To assess changes in chromatin accessibility, we used ATAC‐seq, where accessible loci are identified by the accumulation of DNA sequencing reads (called peaks). The normalized read count within a peak provides a measure of accessibility. The normalized peak profile of samples was subjected to hierarchical clustering and principal component analysis (PCA) (Figure S4a,b). The hierarchical clustering tree and PCA plot show that ATAC peak profile clustered samples by day rather than by donors, with PC1 (90.3% variance) effectively distinguishing unstimulated from day 7 samples (Figure S4a,b).

### 3.6 Most changes in chromatin accessibility are in enhancer regions

**FIGURE 2:**
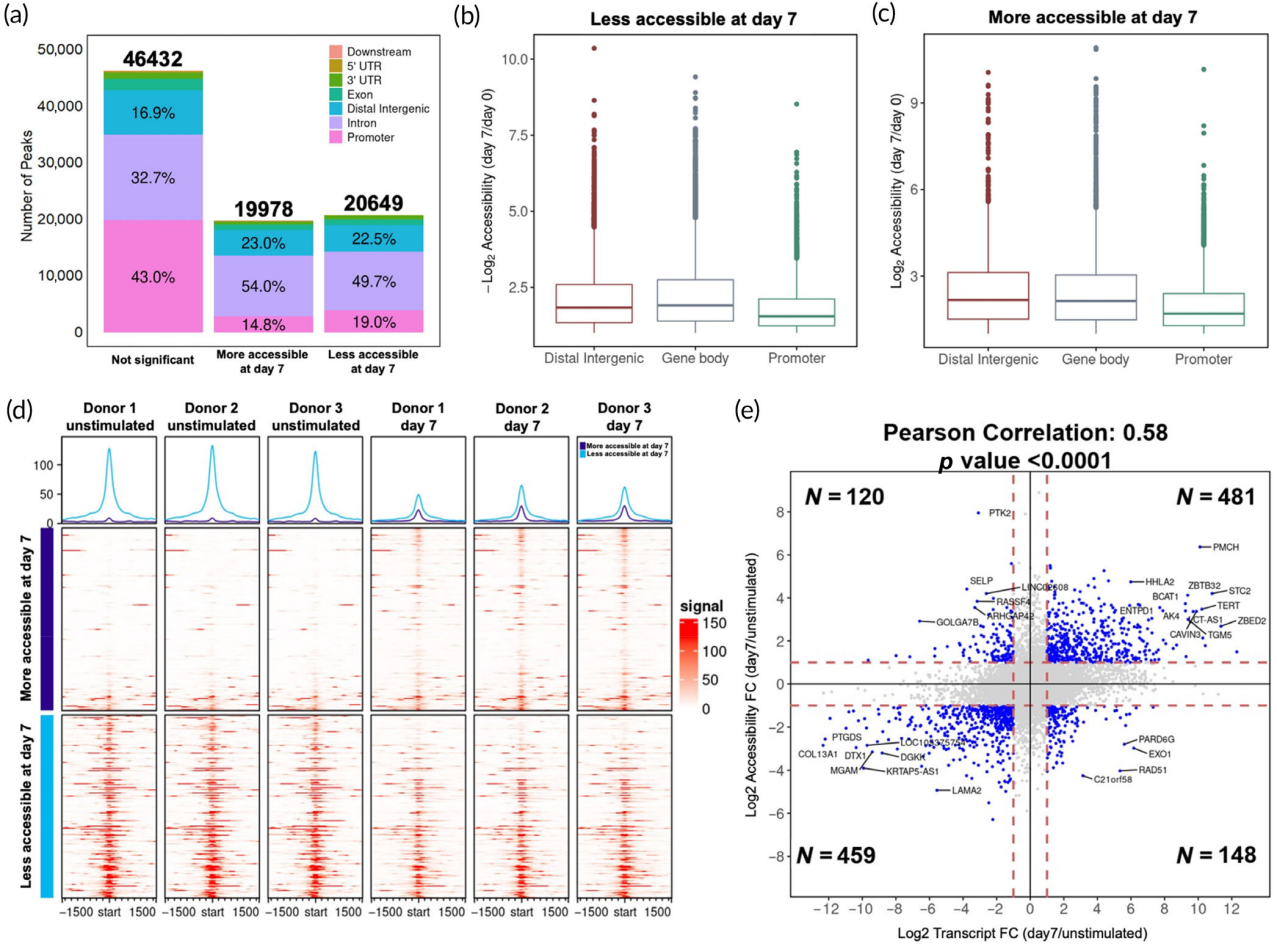
Changes in chromatin accessibility landscape of activated NK cells. (a) Distribution of ATAC peaks annotated to different genome regions and grouped by direction of accessibility changes between day 7 and unstimulated samples. The percentage of the top three genomic regions is shown. (b, c). The fold change in normalized peak signal for differentially accessible peaks (more and less accessible at day 7) and annotated to distal intergenic, promoter, and gene body (intron, exon, 5′ UTR, 3′ UTR). Whiskers extend 1.5 times the inter‐quartile range. (d) ATAC signals in the vicinity of the peak summits for unstimulated and day 7 samples of all three donors. The top 400 differentially accessible peaks with gain in accessibility and the top 400 peaks with reduction in accessibility were used for the plot. Shown are average signals (top row) and heatmaps (bottom rows). The top panel displays the average signal around the peak summits (blue: day 7; purple: unstimulated). The accompanying heatmaps (second and third panel) depict the normalized read density profiles around the summit of peaks, where each horizontal line represents one peak, and peaks are sorted based on log_2_FC (accessibility) in ascending order from top to bottom. (e) Fold change of accessibility of peaks annotated to promoter regions are plotted against the fold change in transcript abundance of the corresponding gene. The number of genes with |log_2_Accessibility FC| > 1 and |log_2_Transcript FC| > 1 for each quadrant is shown. The Pearson correlation values and statistics are shown at top of the plot.

Using the ChIPseeker package, we annotated the peaks based on their distance to the TSS of the closest gene. Peaks within 1000 bp upstream or downstream of a TSS were annotated as promoter, but those overlapping with genes were assigned based on their overlap with exon, intron, 5′UTR, and 3′UTR. Peaks over 1000 bp away from any TSS were labeled as distal intergenic. Using a fold change of 2 and adj. *p*‐value < 0.05 as criteria, about 23% of all peaks became more accessible and 24% less accessible on day 7 (Figure 2a). Notably, more than 68% of the differentially accessible peaks were in distal intergenic or intronic regions, but a majority of nondifferentially accessible peaks were associated with promoters (Figure 2a). Among the differentially accessible peaks, those associated with distal intergenic and gene body regions (intron, exon, 5′UTR, 3′UTR) have higher fold change than those associated with promoter regions (Figure 2b,c). As enhancers are often located in distal intergenic and intronic regions, the results suggest that enhancer regions have a higher likelihood to change accessibility in activation than the promoter regions.

### 3.7 Feeder cell and cytokine activation opens up inaccessible chromatin regions

Figure 2d visually depicts ATAC signals in the vicinity of the summit of peaks for both unstimulated and day 7 samples from all three donors. Peaks with higher accessibility at day 7 (purple color), mostly consist of regions with extremely low accessibility when unstimulated, suggesting they were initially inaccessible but became accessible by day 7. On the other hand, most peaks that were more accessible in unstimulated cells showed a decrease in accessibility by day 7, rather than becoming inaccessible. Thus, activation with feeder cells and IL‐2 opens several previously inaccessible chromatin regions in NK cells while reducing accessibility in some other regions.

### 3.8 Positive correlation between transcript levels and accessibility changes in activated NK cells

We compared the fold changes of accessibility with the fold changes of transcript levels of corresponding genes (Figure 2e). Peaks annotated to the promoter regions of genes were assessed. A positive correlation was observed between promoter accessibility changes and transcript alterations. More genes exhibited changes in both promoter accessibility and transcript abundance in the same direction than opposite direction.

### 3.9 Key TFs have high variability in binding motif accessibility

**FIGURE 3:**
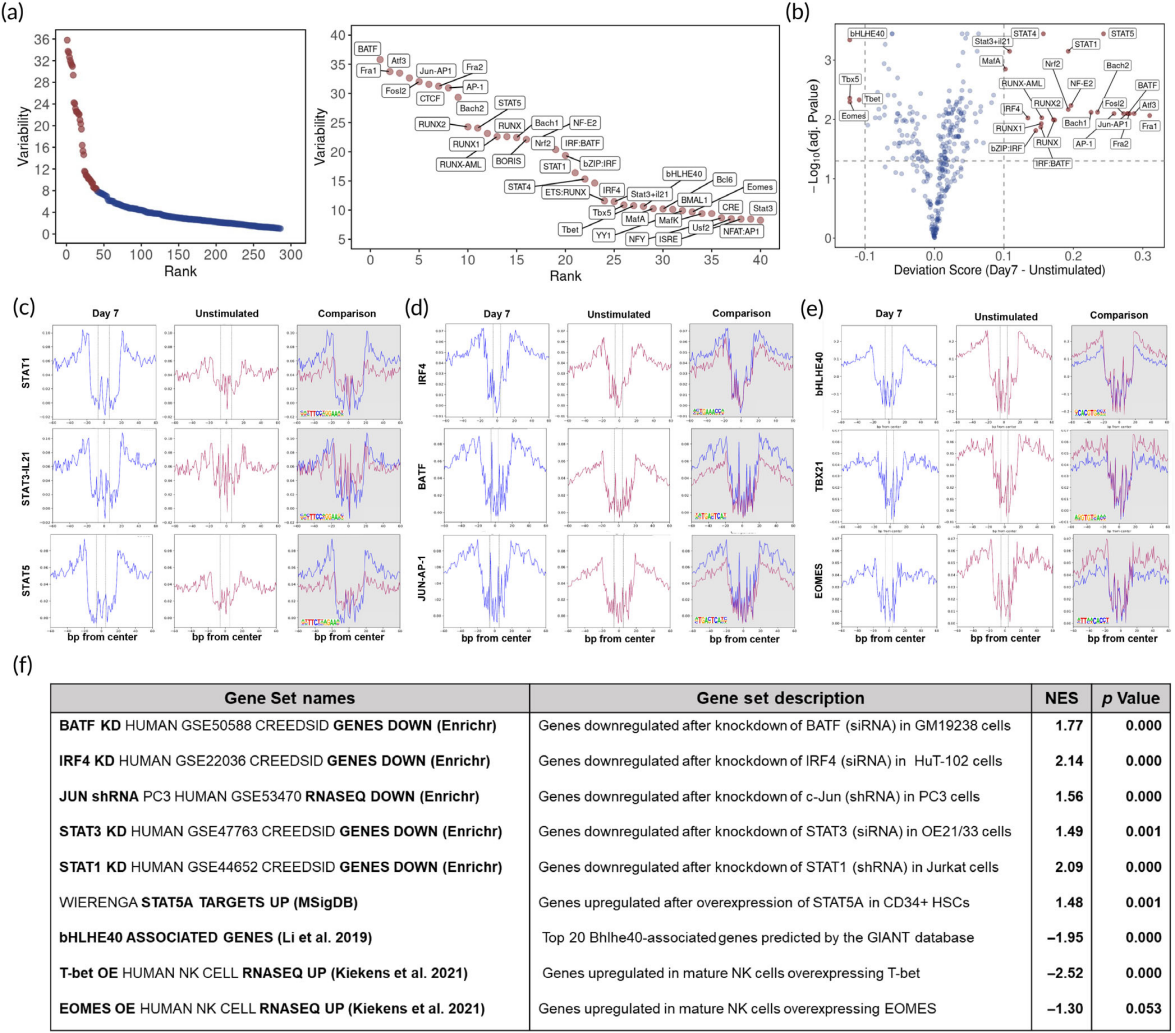
Key TFs which impart large changes in binding activity on their TFBMs were identified. (a) Using chromVAR, TFs are ranked based on the standard deviation (variability) of the Z‐score of their TFBM. The TFs above the inflection point are shown in red and labeled in the zoomed‐in plot. (b) A volcano plot of –log_10_ (adj. *p* value) against the bias‐corrected deviation score of TFBMs, illustrating TFs which elicit significant changes in their TFBM accessibility in the positive or negative direction. The horizontal dashed line indicates adjusted *p* value = 0.05, and the vertical dashed lines show differences in deviation scores equaling 1 and −1. (c–e) Footprint plots of aggregated footprint signals of peaks centered around the consensus TFBM. The consensus logos are shown in the comparison plots. The dashed lines mark the border of motif site for each TF. Three plots for each TF represent the aggregated footprint signal for day 7, unstimulated, and the combined conditions for comparison. (f) TF target gene sets enriched in GSEA. Transcriptome of day 7 and unstimulated were used. The gene sets comprised of the target genes of each TF were retrieved from various databases and studies. The normalized enrichment score (NES) and *p* value for each gene set are also shown.

TFs exert their transcriptional control on downstream genes by binding to their TFBMs. Upon binding, TFs alter the chromatin accessibility around their binding sites. Using chromVAR,^23^ TFs were ranked based on the standard deviation of their TFBM accessibility Z‐score across the samples, referred to as variability (Figure 3a). The top‐ranked TFs surpassing the inflection point are highlighted in red. Among these TFs, several belong to TF families that were previously shown to play key roles in NK cell development and immune cell response, including AP‐1, IRF, NFAT, RUNX, STAT, and T‐box. To assess the direction of changes in the accessibility of TFBMs, bias‐corrected deviation scores were plotted (Figure 3b). More TFs exhibited a statistically significant increase in TFBMs accessibility (positive x‐axis values) at day 7, including AP‐1 members (BATF and JUN), STAT members (STAT1, STAT3, STAT5), and IRF‐4. At day 7, a few TFBMs exhibited decreased accessibility, including bHLHE40, and T‐box members (T‐bet and EOMES).

### 3.10 Footprint analysis confirms the alterations in activity of identified TFs

Binding of a TF to a TFBM results in DNA regions overlapping with and adjacent to a TFBM being protected from Tn5 cutting, thereby exhibiting a lower ATAC signal. Conversely, TF binding increases the ATAC signal in regions surrounding the TFBMs. Thus, the footprints around a TFBM exhibit a characteristic pattern of a low‐signal region centered at the TFBM and increasing signal strength as the region extends outward. The footprints of nine TFs are shown in Figure 3c–e. The depth of a footprint is used to infer the binding activity of a TF.

An increase in footprint depth at day 7 was seen for the STAT family (STAT1, STAT3, and STAT5), IRF4, BATF, and AP1:JUN TFs. Conversely, bHLHE40, T‐bet, and EOMES showed a decrease in the footprint depth, implying fewer binding events of these TFs to their motifs at day 7. These results are in agreement with the chromVAR analysis. The transcript expression of these TFs is plotted in Figure S5a–c. Overall, these TFs are expressed in NK cells, which is necessary for their differential binding activity.

### 3.11 Target gene sets of the key TFs are enriched in Gene Set Enrichment Analysis

TFBM accessibility changes and footprint analyses revealed increased binding of some key TFs and reduced binding of others at day 7. We examined whether the changes in TF binding to TFBMs resulted in transcriptional changes in the target genes of TFs. The target genes of different TFs were retrieved from MSigDB^48^ or EnrichR^49^ databases except for EOMES, T‐bet, and bHLHE40, which were compiled from recently published studies.^50^, ^51^ These TF target gene sets were used in GSEA analysis of the transcriptome to examine whether TFs identified in ATAC‐seq analyses also exhibit enrichment in the gene sets of their target genes.

The gene sets representing TF target genes were enriched, as illustrated in Figures 3f and S5d. Their direction of transcriptional change, as indicated by the positive or negative value of NES, was the same as the ATAC‐seq analyses. This indicates that the changes in the binding of TFs to TFBMs contribute to the differential gene expression of NK cells at day 7.

### 3.12 Dynamics of super‐enhancers (SEs) in NK cells

SEs represent genome regions that are exceedingly accessible and transcriptionally active.^25^ They regulate the expression of genes determining cell identity and can undergo dynamic alterations during cell differentiation, development, and disease to facilitate cell state transitions.^20^, ^25^, ^52^ Although the conventional method of identifying SEs involves using histone H3K27ac ChIP‐seq data, ATAC‐seq has also been employed in recent years to identify potential SEs.^18^, ^19^

**FIGURE 4:**
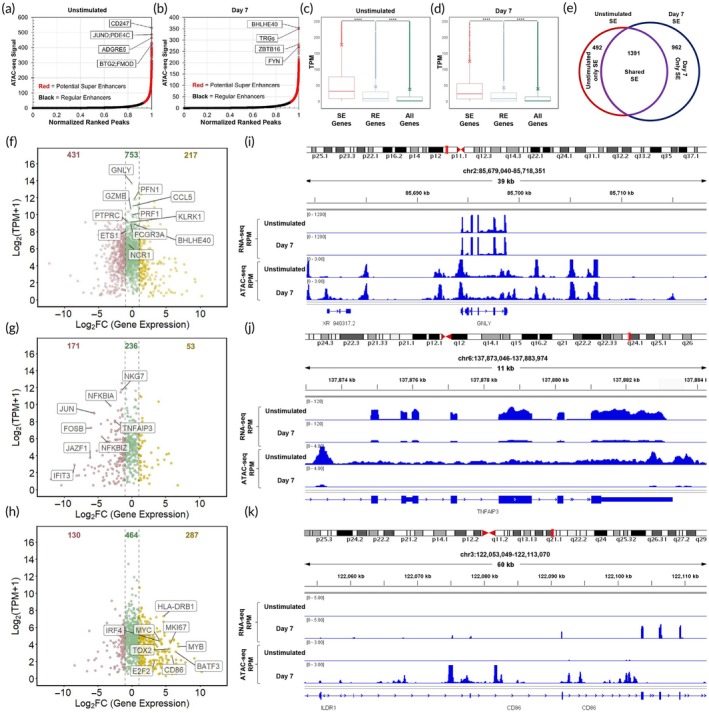
SEs in unstimulated and activated NK cells are identified. (a, b) ATAC‐seq peaks in unstimulated and day 7 NK cells, ordered by normalized read coverage after background subtraction, are plotted against normalized ranked peaks. The stitched peaks designated as SEs are located above the inflection point. The top SE peaks, based on their ATAC‐seq signal, are labeled. (c, d) Boxplots showing gene expression of SE associated genes, regular enhancer associated genes, and all other genes in unstimulated and day 7 NK cells. Whiskers extend 1.5 times the inter‐quartile range, and “x” represents the mean. (e) A Venn diagram illustrates the count of SEs exclusively present in unstimulated NK cells, those shared between unstimulated and day 7, and those exclusively found in day 7 NK cells. (f–h) Average abundance (displayed by Log_2_ (TPM + 1)) of genes assigned to SEs is plotted against the Log_2_fold change in gene expression for “shared”, “unstimulated only”, and “day 7 only” SEs, respectively. The vertical dashed lines show FC = 2 and FC = –2. Upregulated genes are shown in yellow, downregulated are shown in pink, and those that are not significantly changed in green. Some of the genes discussed in the manuscript are labeled. (i) Local genome view of representative shared SE with highly active and stable transcriptional activity. (j) Local genome view of a representative day 0 only SE with unstable transcriptional activity. (k) Local genome view of a representative day 7 only SE with unstable transcriptional activity. **** indicates *p* value <0.0001.

Stitched peaks were ranked by their ATAC‐seq signal values, and the inflection point was used to distinguish regular enhancers from SEs (Figure 4a,b), as described by Whyte et al.^25^ The SEs, shown in red, were assigned to the closest genes using Homer. A total of 1883 stitched peaks were identified as SEs in unstimulated cells, and 2353 SEs were identified at day 7. The expression of genes linked to SEs, regular enhancers, and all other genes with no enhancer associations are shown in Figure 4c,d. In both unstimulated and day 7 cells, genes close to SEs exhibited significantly higher transcript expression compared to those near regular enhancers or other genes, indicating a positive impact of SEs identified by ATAC‐seq on the transcriptional activity of linked genes.

### 3.13 Stable and condition‐dependent SEs in NK activation

SEs were classified into three groups: those which were SEs both in day 7 and unstimulated NK cells (“shared”), those found only at day 7 (“day 7 only”), and those found only in unstimulated cells (“unstimulated only”). About half (1391) of the identified SEs were “shared,” and 962 were “day 7 only,” and 492 were “unstimulated only” (Figure 4e).

To assess the contribution of TFs to the transition from non‐SE in unstimulated cells to SE state on day 7, we employed a motif enrichment tool, i‐cisTarget,^53^ to identify the enrichment of TFBMs in the “day 7 only” SEs. Several TFs identified in the previous section to be more active at day 7, including STAT1, STAT3, STAT5A, IRF4, BATF, and IRF:BATF, exhibited enriched TFBMs in “day 7 only” SEs (Figure S6a), suggesting the potential involvement of these TFs in the establishment of multiple “day 7 only” SEs.

### 3.14 Changes in transcriptional activity of SE‐associated genes

Although SEs exert a strong influence on the transcriptional activity of their associated genes, the transcriptional regulation of a gene involves multiple other regulatory elements, and acquisition or loss of SE status is not necessarily correlated with transcript level changes. This is illustrated by plotting fold changes in transcript levels against average transcript expression levels for genes in each SE group (Figure 4f–h). About half of the genes in all groups (46%–51%) had fold changes of less than 2 (depicted in green and within two vertical dashed lines). The “unstimulated only” group had more downregulated (171) compared to upregulated (53) genes, but the “day 7 only” group had more genes upregulated (287) than downregulated (130).

Many “shared” SE‐associated genes (shown in green color) play critical roles in NK cell identity and function. Some of these genes include cytolytic molecules like *GNLY* (granulysin), *PRF1* (perforin), and *GZMB* (granzyme B) and cytolytic regulators like *PFN1* (profilin 1). Some other labeled genes such as *PTPRC* (CD45), *FCGR3A* (CD16a), *NCR1* (NKp46), and *KLRK1* (NKG2D) encode cell surface proteins involved in initiating the effector function of NK cells. Additional genes in this group include chemokines such as *CCL5* and TFs such as *BHLHE40* and *ETS1* that are important in NK cell function.

Several genes in the “unstimulated only” group that exhibit a decrease in transcript level at day 7 are related to immune response and are labeled in Figure 4f. Among these genes, *JUN* (c‐Jun) and *FOSB* (FosB) are TFs downstream of natural cytotoxicity receptor activation. *NFKBIZ* (IκBζ), *NFKBIA* (IκBα), *JAZF1*, and *TNFAIP3* serve as inhibitors of NF‐кB signaling and inflammation. *NKG7* is involved in immunological synapse formation and granule exocytosis, while although *IFIT3* plays crucial roles in antiviral innate immunity.

Examples of “day 7 only” SEs with increased gene expression at day 7 include *CD86* and *HLA‐DRB1*, which are expressed on activated NK cells and have the potential to interact with other immune cells. Moreover, this group encompasses several TFs that regulate cell cycle progression and metabolic reprogramming, such as *E2F2*, *MYB*, *MYC*, *BATF3*, and *IRF4*. Among other genes within this SE category are *CDK6* and *MKI67*, which are implicated in cell proliferation, along with *TOX2*, a TF crucial in NK cell development.

An illustration of an SE region encompassing *GNLY*, an antimicrobial agent in NK cell cytotoxic granules,^54^ is shown in Figure 4i, where both RNA‐seq and ATAC‐seq signals were high in unstimulated and day 7 samples. The SE region overlapping with *TNFAIP3*, an anti‐inflammation gene, had decreased ATAC signal and diminished transcript abundance in day 7 NK cells (Figure 4j). The SE region containing *CD86*, an activating receptor on NK cells whose expression increases after NK cell stimulation,^55^ is shown in Figure 4k. Both ATAC‐seq and RNA‐seq signals of the shown region are increased significantly at day 7 compared to unstimulated NK cells.

## 4 DISCUSSION

Feeder cells are widely used in the expansion of NK cell products.^8^ Feeder cell activation also makes NK cells more susceptible to viral transduction for cell engineering, as unstimulated NK cells are highly resistant to transduction.^56^ In this study, we characterized the transcriptome and chromatin state of NK cells before and after activation with feeder cells and IL‐2 and identified genome regions that are highly accessible before and after activation and are likely to be favorable for gene insertion to gain stable and high levels of gene expresion.^18^

### 4.1 Interplay between accessibility alterations and transcript changes

Activation led to significant changes in gene expression and chromatin accessibility in NK cells. A large proportion of genes showed differential expression, particularly those involved in histone modifications and metabolic pathways, which provide histone‐modifying enzymes and metabolite precursors to remodel chromatin. Additionally, several genes encoding pioneer TFs such as *MYB, IRF4*, and *RUNX3* were differentially expressed, which can alter chromatin accessibility by displacing nucleosomes. Previous studies have reported that immune cell stimulation activates pioneering TFs and results in significant remodeling of the chromatin.^57^, ^58^, ^59^ Chromatin accessibility increases, specifically in regions that were previously inaccessible, can impact the recruitment of TFs to new genome regions, thereby influencing the transcriptional activity of genes in different pathways including metabolic pathways and histone modification.^60^ For instance, *IRF4*, a pioneer TF associated with a “day 7 only” SE, exhibits significantly higher transcript expression at day 7. Its TFBM accessibility also increases, implying its enhanced binding events to genome regions close to its target genes. IRF‐4 directly regulates the expression of multiple genes including itself and *MYC*.^28^, ^29^ Transcript levels of *MYC* are highly increased at day 7, and it is associated with a “day 7 only” SE. As a global amplifier of active genes, c‐Myc plays a key role in upregulating glycolysis and TCA cycle genes and thereby influences histone modification by changing metabolite levels.^61^ The bidirectional interplay between changes in the transcriptome, histone modifications, metabolic pathways, and chromatin accessibility highlights the complex regulatory mechanisms governing NK cell function and their response to external stimuli. TFs emerged as central players in orchestrating these regulatory processes in immune cell activation and response.^58^

### 4.2 Various TFs are involved in NK cell activation

We examined the binding activity of TFs in NK cells through binding motif accessibility, footprint, and gene set enrichment of target transcripts. The AP‐1 family of TFs, including c‐Jun and BATF, showed increased TFBM accessibility in activated NK cells. AP‐1, activated by natural cytotoxicity receptor engagement in NK cells, has been implicated in memory NK and T‐cell formation.^15^, ^34^, ^60^, ^62^, ^63^ Studies suggest that c‐Jun and BATF overexpression in CAR‐T cells enhances antitumor functions and counters exhaustion, emphasizing the importance of AP‐1 in regulating lymphocyte function.^64^, ^65^

The STAT family also exhibited a significant increase in footprint depth and TFBM accessibility. JAK–STAT signaling is pivotal in cytokine stimulation of NK cells, with IL‐2 primarily activating STAT5, and to a lesser extent, STAT3 and STAT1. In contrast, IL‐21 predominantly activates STAT3, minimally involving STAT1 and STAT5 (Figure S6b).^66^ Previous work has demonstrated potential interactions between STAT and AP‐1 TFs in activated NK cells.^67^

IRF‐4, more active in day 7 NK cells, is downstream of T‐cell receptor (TCR) signaling in T cells and regulates aerobic glycolysis.^28^ In activated T cells, IRF4 and AP‐1 bind cooperatively, and the level of AP‐1/IRF4 complexes correlates with the transcriptome response downstream of TCR signaling.^68^ STAT3 can also interact with IRF4 to control the expression of IL‐21‐dependent genes in T cells.^36^, ^69^ Cooperation of different TFs including STATs, IRF4, BATF, c‐Jun, etc., has been proposed to be involved in the formation of SEs in activated T cells.^60^ Interestingly, SEs that are unique to activated NK cells (“day 7 only SEs”) are also enriched in TFBMs for STAT3, IRF4, BATF, and STAT5, implying the participation of these TFs in the formation of some of the SE regions.

### 4.3 SEs in NK cells

Using ATAC‐seq data, we identified SEs in unstimulated and day 7 NK cells. Within the SEs, there was a group present in both unstimulated and day 7 cells (“shared”). Among “shared” SEs, a subset displayed high transcriptional activity with minimal changes in gene expression between the two time points. Several genes belonging to this category of SEs encode proteins that are crucial for NK cell function and identity. Furthermore, these transcriptionally stable “shared” SEs could be favorable for the insertion of transgenes requiring sustained transcription following activation such as CARs. Previous studies have shown that the expression of CARs in T cells or macrophages could be unstable and condition‐dependent, highlighting the importance of stable transgene expression.^70^, ^71^, ^72^

Additionally, certain SEs were exclusively detected either on day 7 or in unstimulated samples. The emergence or disappearance of these SEs appeared to be contingent upon the activation of NK cells, suggesting that the genes under their regulatory influence may play pivotal roles in NK cell activation. Among the SEs only identified on day 7, several associated genes were implicated in cell cycle progression and metabolic reprogramming, and SEs found only in unstimulated samples were linked to genes associated with the inflammation and natural cytotoxicity response in NK cells.

## 5 CONCLUSIONS

Analyzing the transcriptome and open chromatin state of feeder cell and IL‐2‐activated NK cells yields valuable insights into the functional transition of NK cells. Identifying the key TFs orchestrating the transition to a highly proliferative state informs strategies for improving NK cell function and expansion. Potential strategies involve providing stimulation signals upstream of identified TFs or overexpressing these TFs to improve NK cell proliferation and persistence in vivo. In addition, the identification of shared SEs with stable transcriptional activities guides the selection of regions suitable for transgene insertion with potential long‐term gene expression stability.

## AUTHOR CONTRIBUTIONS

**Pedram Motallebnejad:** Conceptualization; formal analysis; data curation; investigation; methodology; visualization; writing – original draft; writing – review and editing; software. **Zion Lee:** Formal analysis; data curation; investigation; methodology; visualization; writing – review and editing. **Jennifer L. One:** Conceptualization; data curation; methodology; writing – review and editing. **Frank Cichocki:** Conceptualization; resources; supervision; writing – review and editing. **Wei‐Shou Hu:** Conceptualization; resources; supervision; writing – review and editing. **Samira M. Azarin:** Conceptualization; supervision; resources; writing – review and editing; funding acquisition.

## FUNDING INFORMATION

This work was supported by NSF CBET‐1845366 (S.M.A) and NIH R01 HL155150 (F.C.).

## CONFLICT OF INTEREST STATEMENT

The authors declare no conflicts of interest.

## Supporting information


**Data S1.** Supporting information.

## Data Availability

The data sets used and/or analyzed in the current study are available from the corresponding authors upon reasonable request. ATAC‐seq and RNA‐seq data generated in this manuscript will be publicly available from the Gene Expression Omnibus (GSE268340; GSE268341) from May 24, 2025.
