# Supplementary material for: Integrated ATAC‐seq and RNA‐seq analysis identifies key regulatory elements in NK cells activated with feeder cells and IL‐2
Source: Bioeng Transl Med. 2025 Feb 26;10(3):e10747. doi: 10.1002/btm2.10747 (PMC12079432; doi:10.1002/btm2.10747)
Supplement: Supplementary file 1 — Data S1. Supporting information. [file BTM2-10-e10747-s001.pdf]

Supplementary Information

Integrated ATAC-seq and RNA-seq analysis identifies key regulatory elements in NK cells activated with feeder cells and IL-2

Pedram Motallebnejad, Zion Lee, Jennifer L. One, Frank Cichocki, Wei-Shou Hu, Samira M. Azarin

Supplemental Materials:

Supplemental Figures S1-S6

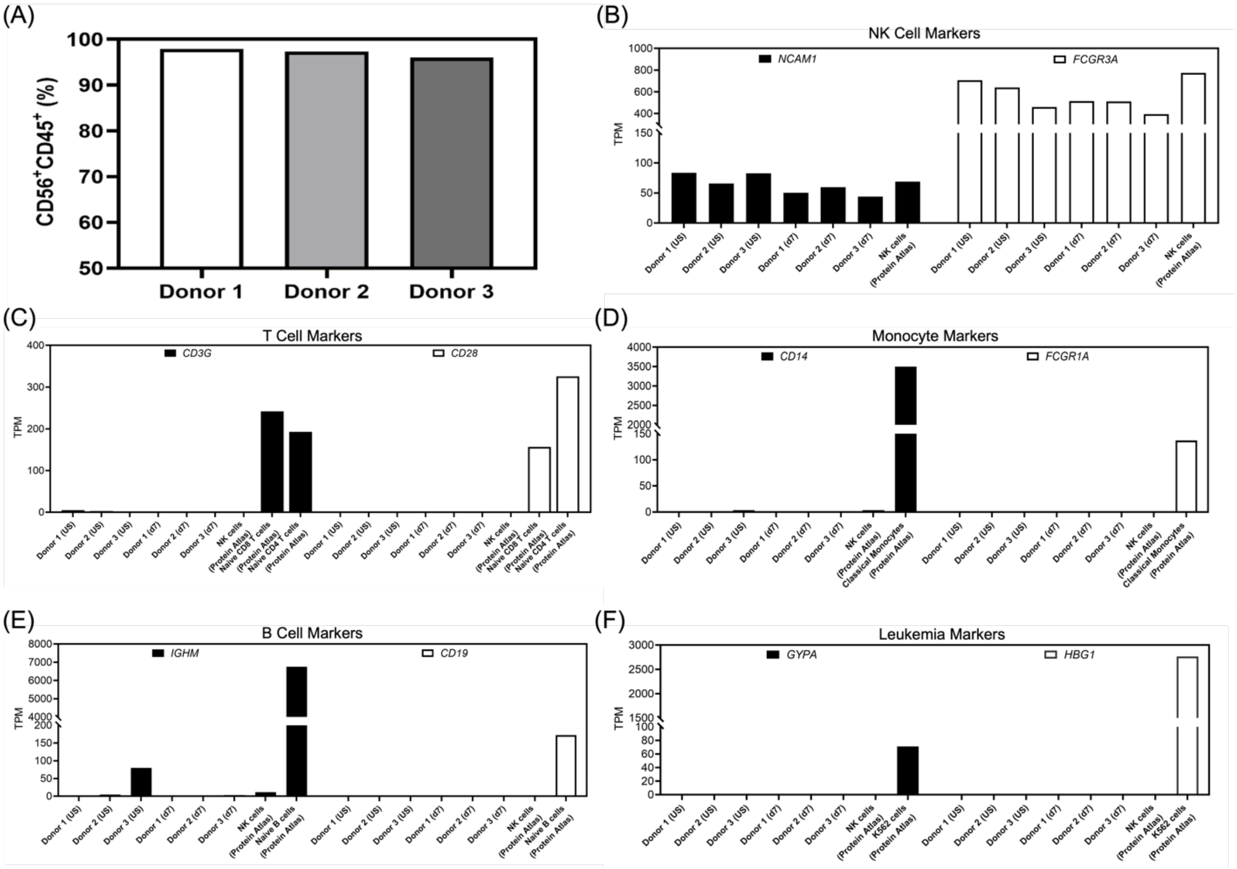

Figure S1. Purity of NK cell samples used for RNA-seq and ATAC-seq. (A) Percentage of CD45+CD56+ cells in day 7 samples as determined by flow cytometry. (B-F) TPM values for markers of NK cells (B), T cells (C), monocytes (D), B cells (E), and leukemia cells (F) in each sample, compared to the values reported in the Protein Atlas for the respective PBMC types and NK cells.

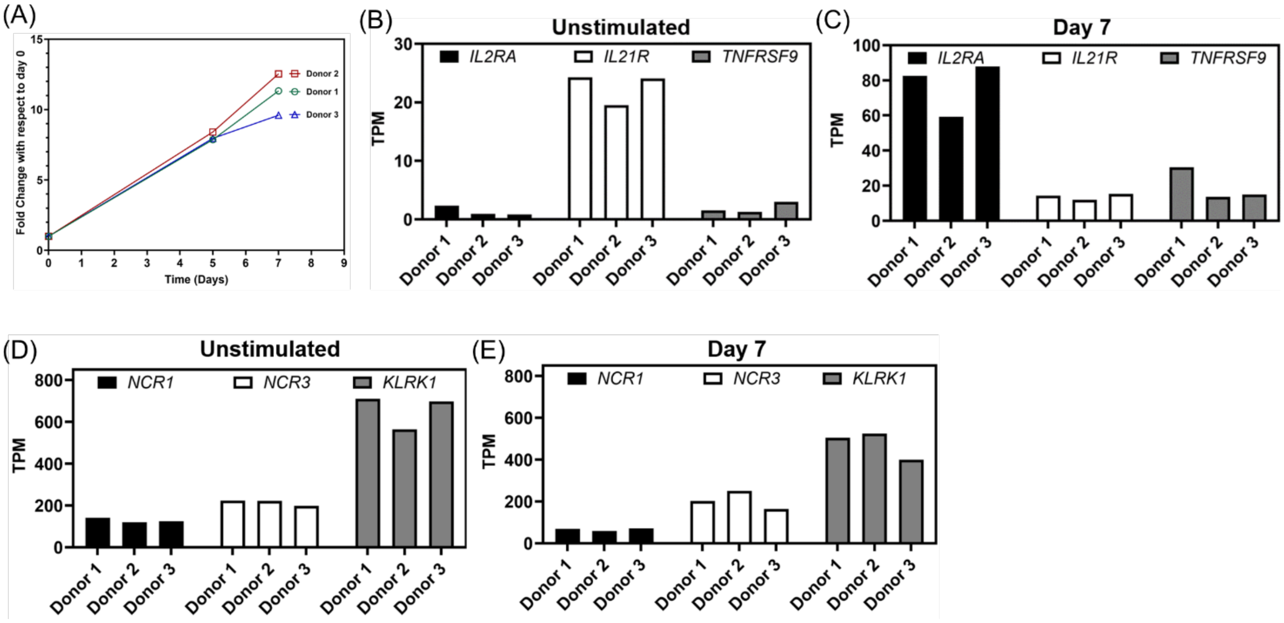

Figure S2. Comparison of different donors used for RNA-seq and ATAC-seq. (A) Fold expansion of different donors relative to day 0, based on the total viable cell number ratios at the indicated day. (B-C) TPM values of selected cytokine receptors in unstimulated (B) and day 7 (C) samples for individual donors. (D-E) TPM values of selected activating receptors in unstimulated (D) and day 7 (E) samples for individual donors.

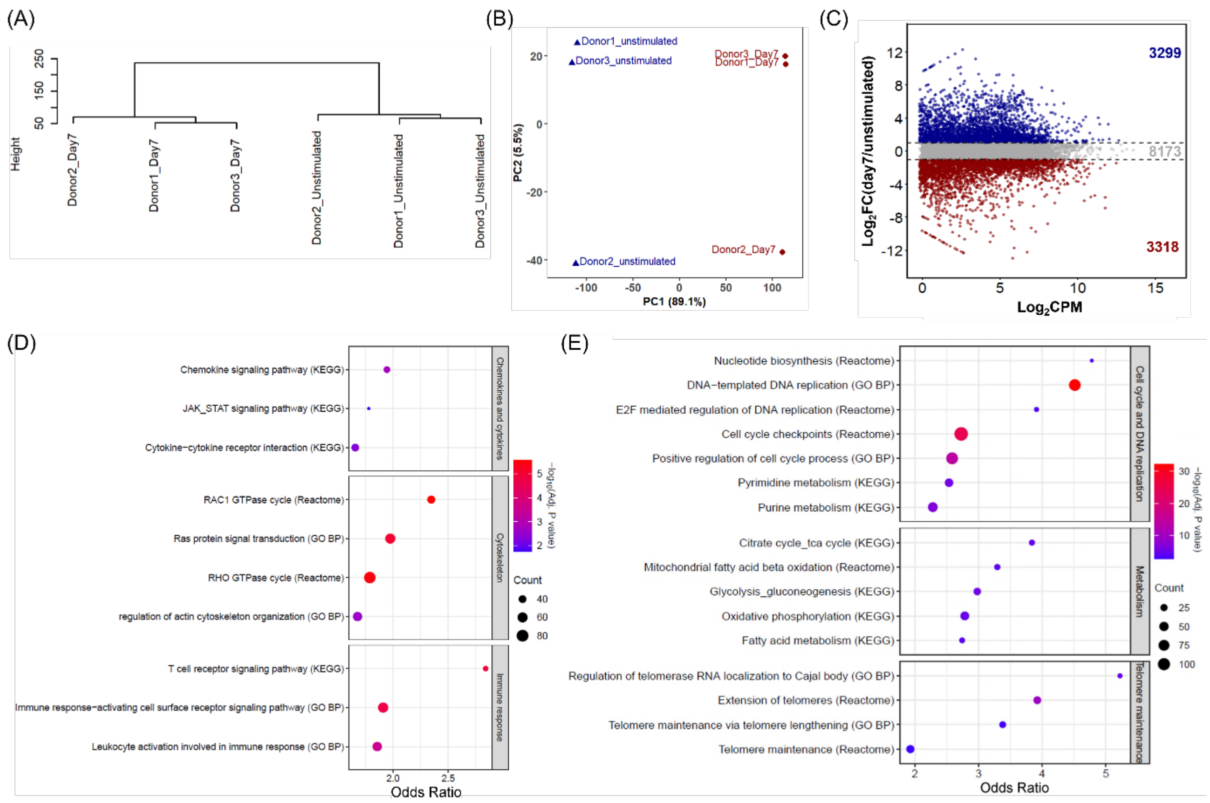

Figure S3. RNA-seq analysis of NK cell activation with feeder cells. (A) Sample clustering tree of RNA-seq results showing stronger clustering of samples by day than by donor. (B) PCA of RNA seq results showing the importance of PC1 in differentiating unstimulated from day 7 samples. (C) MA plot showing the Log_2 fold change in transcript abundance versus the average expression level shown by Log_2CPM. Upregulated genes Log_2FC(d7/unstimulated) >1 and adj. P value <0.05) are labeled in blue and downregulated genes Log_2FC(d7/unstimulated) <-1 and adj. P value <0.05) are shown in red. The dashed lines indicate fold change of 2. The number of genes that are upregulated, downregulated, and not changed are shown in the plot. (D-E) Overrepresentation analysis results on downregulated DEGs at day 7 (D) and upregulated DEGs at day 7 (E) using Gene Ontology (GO) Biological Processes (GO BP), KEGG, and Reactome data sets. The values corresponding to $-log_{10}$(Pvalue) are shown by different colors, and the number of genes in each group is indicated by the size of the circle.

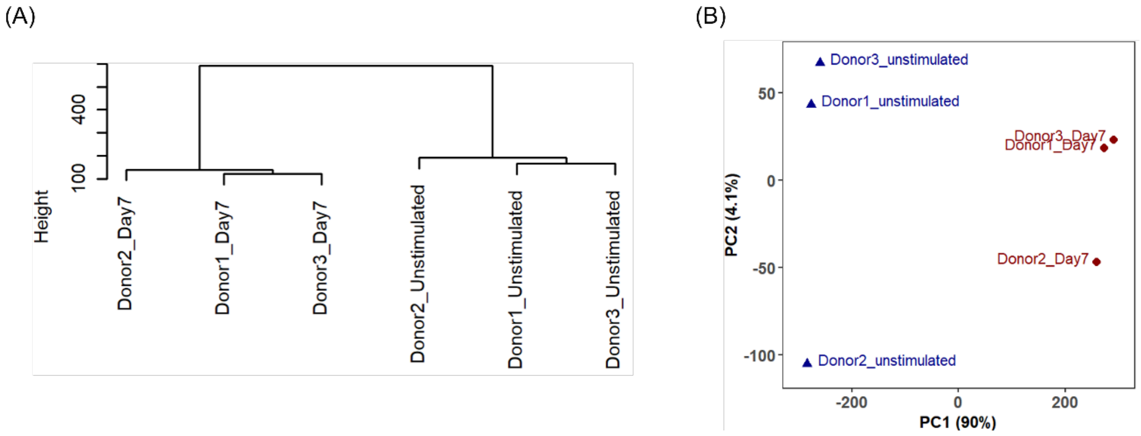

Figure S4. ATAC-seq data analysis of NK cell activation with feeder cells. (A) Hierarchical clustering of normalized ATAC peak reads. Samples were strongly clustered by day rather than by donor. (B) PCA of ATAC-seq shows effective differentiation of unstimulated from day 7 samples by PC1.

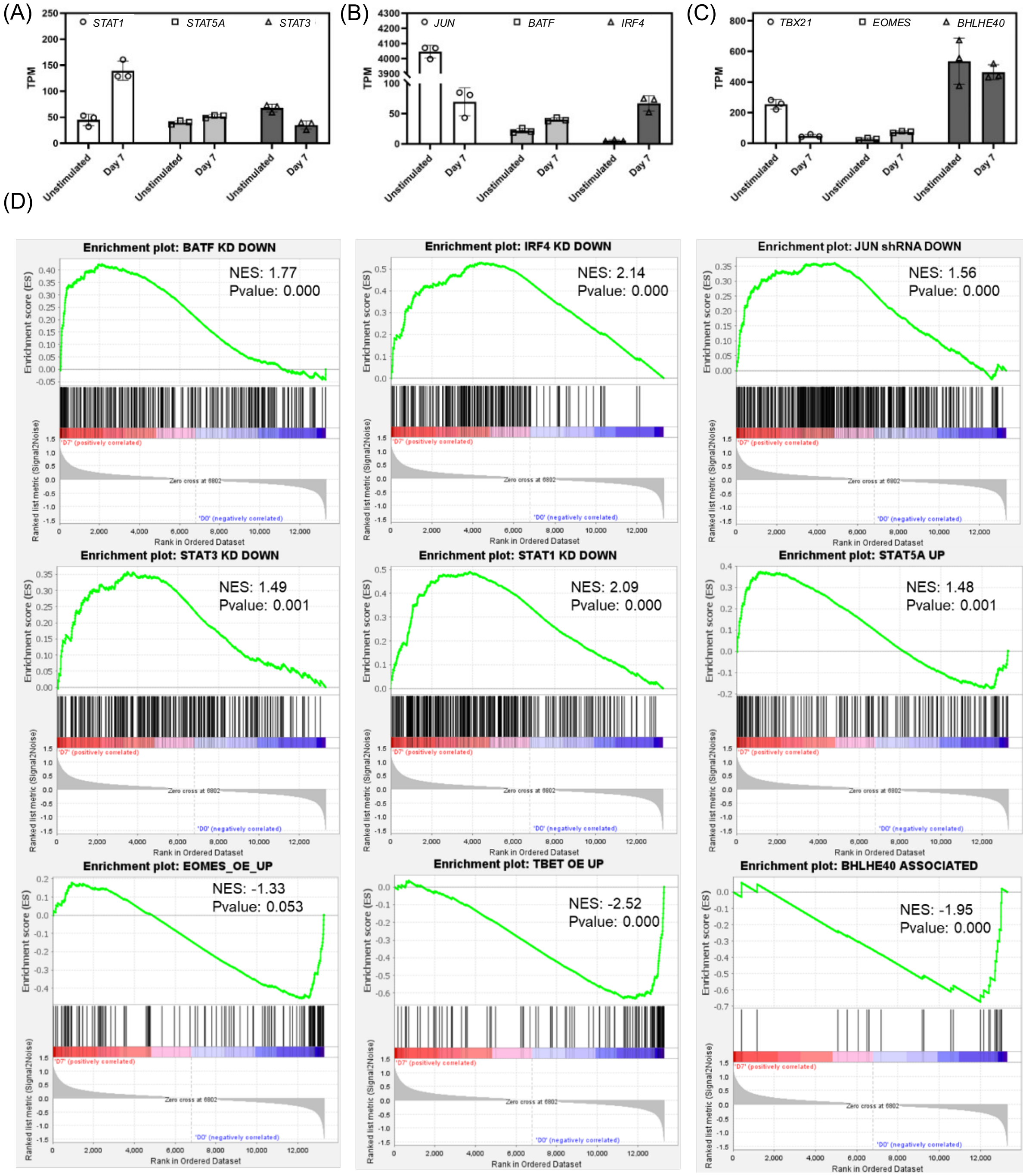

Figure S5. TF expression and TF target gene enrichment in unstimulated and day 7 samples. (A) Transcript expression levels of STAT family members in unstimulated and day 7 samples. (B) Transcript expression levels of JUN, BATF, and IRF4 in unstimulated and day 7 samples. (C) Transcript expression levels of TBX21, EOMES, and BHLHE40 in unstimulated and day 7 samples. (D) GSEA plots corresponding to the data presented in Figure 3G.

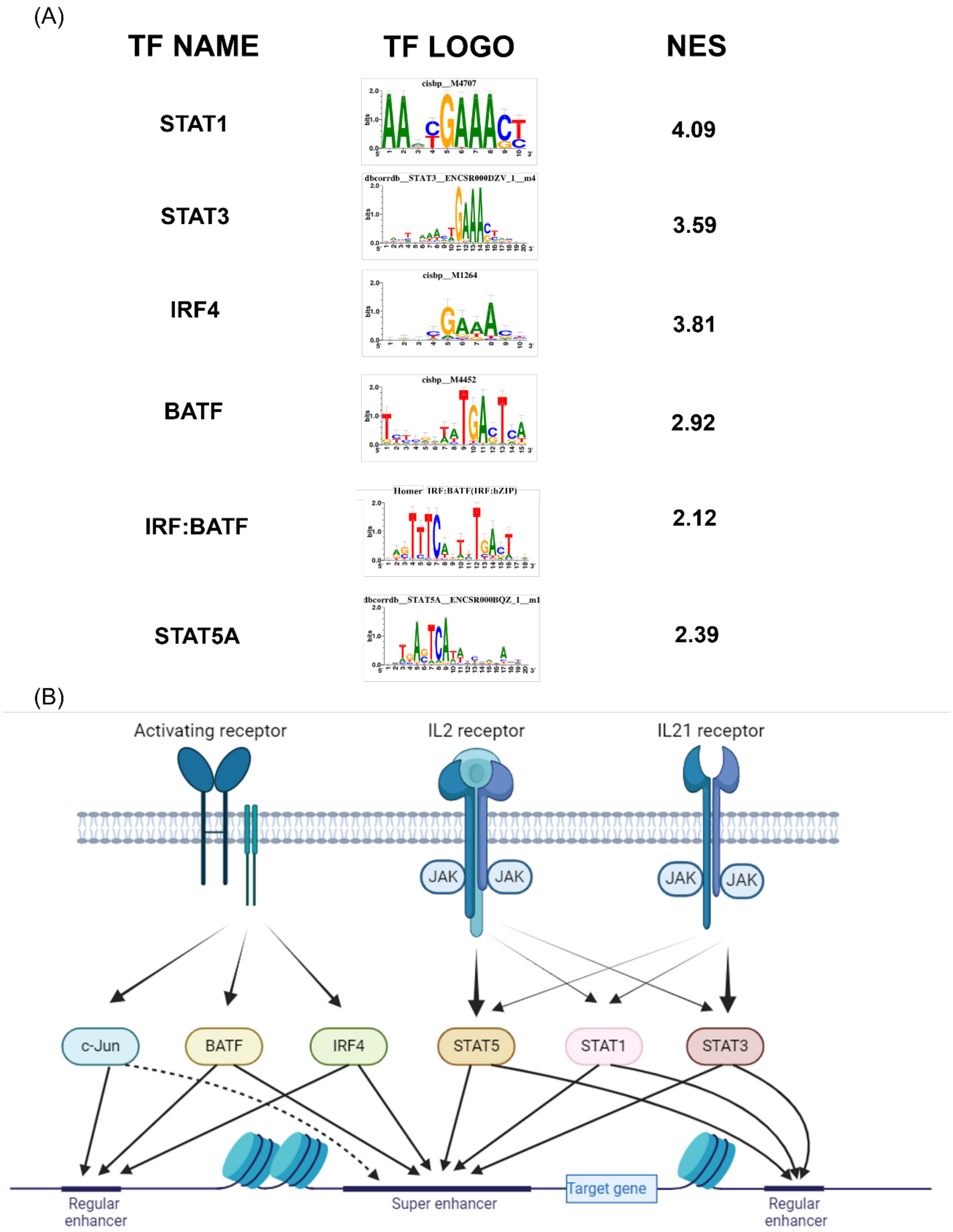

Figure S6. TFs involved in the formation of SEs at day 7. (A) Motif enrichment results for day 7 only SEs using i-cisTarget. Normalized enrichment score (NES) and the TF motif logo are included in the results. (B) A schematic showing the potential interactions between different TFs in the formation of “day 7 only” SEs.
